# Improving the usability and archival stability of bioinformatics software

**DOI:** 10.1186/s13059-019-1649-8

**Published:** 2019-02-27

**Authors:** Serghei Mangul, Lana S. Martin, Eleazar Eskin, Ran Blekhman

**Affiliations:** 1 Department of Computer Science, University of California Los Angeles, 580 Portola Plaza, Los Angeles, CA 90095 USA; 2 Institute for Quantitative and Computational Biosciences, University of California Los Angeles, 611 Charles E. Young Drive East, Los Angeles, CA 90095 USA; 3 Department of Human Genetics, University of California Los Angeles, 695 Charles E. Young Drive South, Los Angeles, CA 90095 USA; 4 Department of Genetics, Cell Biology and Development, University of Minnesota, 321 Church St SE, Minneapolis, MN 55455 USA; 5 Department of Ecology, Evolution, and Behavior, University of Minnesota, 100 Ecology Building, 1987 Upper Buford Cir, Falcon Heights, MN 55108 USA

## Abstract

Implementation of bioinformatics software involves numerous unique challenges; a rigorous standardized approach is needed to examine software tools prior to their publication.

The rapid advancement of genomics and sequencing technologies has led to an overwhelming amount and diversity of new analytical algorithms packaged as software tools [1]. Such computational tools have helped life science and medical researchers analyze increasingly complex data, solve difficult biological problems, and lay groundwork essential for novel clinical translation. Indeed, all phases of sequencing data analysis rely on bioinformatics tools [2], from the initial sequencing of the human genome to modern analyses of high-throughput sequencing data.

With the increasing importance and popularity of computational and data-enabled approaches among biomedical researchers, it becomes ever more critical to ensure that the developed software is usable [3] and the uniform resource locator (URL), through which the software tool is accessible, is archivally stable. Consistently usable and accessible software provides a foundation for the reproducibility of published biomedical research, defined as the ability to replicate published findings by running the same computational tool on the data generated by the study [4, 5]. In addition, the usability—or “user-friendliness”—of software tools is important, and software tools developed in academia are often less user-friendly compared with tools developed in industry settings. Given the abundance of tools released each year, these issues can limit a software tool’s scientific utility. While the scale of this issue in computational biology has yet to be estimated, the bioinformatics community warns that poorly maintained or implemented tools will ultimately hinder progress in “big data”-driven fields [6].

Successfully implementing and widely distributing scientific software involves numerous unique challenges. First, in academia, software tools are developed by small groups comprised of graduate or postdoctoral scholars [7]. These groups are less comprehensively trained and collaborate for shorter durations when compared with software development groups in the industry, where holistic teams of specialists support the long-term maintenance of projects.

In addition, even a stable online presence provides no guarantee of the software tool’s usability, defined as the ability of the user to install and run the tools. Limited software usability and archival stability of computational tools can limit the applicability of the developed software tool and ultimately impair our ability to reproduce the results obtained using the software tools in the original publication. In academia, developers often lack formal training in software engineering, particularly in specialized user experience and cross-platform design. Many computational biology software developers lack the resources to provide a user-friendly interface for installing and running the tools. Developing an easy-to-use tool is further complicated by many tools’ reliance on third-party software that needs to be installed in advance, called “dependencies.” The computational biology field currently lacks a standardized approach for allowing the end user to easily install tools.

Lack of institutional support for software development exacerbates these challenges. Although agencies are interested in novel computational methods, there is inadequate funding for the continual development and maintenance of existing tools [8]. Even widely used tools can suddenly lose the funding that is necessary for long-term maintenance—halting, and even discontinuing, development and availability of the tool. In addition, software development is not incentivized in academic hiring and promotion, processes that focus primarily on publication and funding rather than the development of software tools and resources.

The increasing synergy between computational and wet lab researchers motivated us to perform a survey of the usability and archival stability of omics computational tools and resources [9]. Our empirical analysis of 24,490 omics software resources published from 2000 to 2017 shows that 26% of all omics software resources are not currently accessible through URLs published in the original paper. Despite a strong decline in the percentage of archivally unstable resources from 2000 to 2017, there remain 200 archivally unstable resources published each year during the study period.

Improving the usability and archival stability of computational tools requires a concerted effort among software developers in academia, and our study points at several promising initiatives. We recommend hosting software tools on websites, such as GitHub and SourceForge, designed to host source code. In our study, software tool URLs directing the reader to such online repositories have a high rate of accessibility; 99% of the links to GitHub and 96% of the links to SourceForge are accessible, while only 72% of links hosted elsewhere are accessible. The bioinformatics community has used these web-based services since 2001, and the proportion of software tools hosted on these repositories has grown substantially—among papers included in our study, from 5% in 2012 to 20% in 2017 [9].

Further, our systematic assessment of the installability of published omics software tools suggests several areas of improvement. Among the 99 randomly selected tools included in our analysis, 49% were deemed “difficult to install,” meaning installation required more than 15 min, and 28% of the tools failed to be installed within the 2-h limit, usually due to implementation problems. Moreover, we found that installability impacts the popularity of software tools; successfully installed tools had significantly more citations compared with tools that we were not able to install within 2 h. On average, eight commands were required to install the surveyed tools, while user manuals provide an average of only 3.9 commands. Several surveyed software tools were available via a package manager, which allows the user to consistently automate the installation, upgrade, and configuration. We found that tools available through well-maintained package managers (e.g., Bioconda [10]) were always installable, while tools shipped without package managers were prone to problems in 32% of the studied cases.

As bioinformatics researchers address increasingly complex datasets and problems, our community needs to adopt rigorous and standardized approaches to developing, peer reviewing, and published software packages. Many solutions to archival instability are already available, pragmatic, and analogous to existing practices in digital data archiving. For example, hosting bioinformatics software packages on archivally stable services, such as GitHub or SourceForge, greatly improves the long-term accessibility of omics tools. Solutions to unusable packages are more varied. Developers could create and provide an easy-to-use installation interface capable of downloading and installing any required third-party software packages, known as dependencies. Alternatively, developers can wrap the tools in package managers such as Bioconda [10]. Similar to the unit and integration testing practices in software engineering, an example dataset with a description of the expected results allows the user to verify that the tool was successfully installed and works properly before running the tool on experimental data.

Journals may need to encourage a rigorous, standardized approach to software usability and accessibility by formally taking the issues into account during the peer review process. Reviewers may require that papers describing software tools include relevant items such as installation scripts, test data, and functions that allow automatic checks for the plausibility of installing and running the tool. Journals can provide a complementary top-down strategy to address another growing problem in our field: version control, or maintaining a stable software system that consists of many versions and configurations. For example, forking is a procedure designed to ensure the version of cited code within an article may persist beyond initial publication. Recently, the journal *eLife* took a major step toward improving archival stability by permanently forking published software to GitHub.

We are witnessing an exciting time in bioinformatics. Each year, rapid advances in omics technologies prelude an astonishing number of software tools designed to accommodate increasingly bigger, more complex, and more specialized datasets. Dramatic changes in bioinformatics research and high-throughput computing capabilities can render some tools irrelevant—yet provide context for the development of new tools with superior accuracy. Our study highlights the challenges of producing usable and archivally stable bioinformatics software. The current model of computational biology software development encourages researchers to develop and publish novel tools but does not incentivize the maintenance of existing tools. Moreover, in academia, there is little motivation to develop computational tools that are easy to install and use. Nevertheless, results from our study broadly capture the importance of software stability and usability to the growth of computational biology. Our results provide compelling evidence for the adoption of a concerted effort toward a standardized approach for verifying and archiving software and highlight the need for funding and resources dedicated for the development and maintenance of software tools in the biomedical research community.
